# Patients and Their "Beauty Sleep"

**Published:** 1914-01-31

**Authors:** 


*To the Editor of* The Hospital.

### Patients and their "Beauty Sleep."

*To the Editor of* The Hospital.

Sir,—I have read with interest the correspondence regarding the early washing of patients. On four separate occasions I have been an in-patient at different hospitals for a period of two to five weeks. Personally I never felt the least inconvenience or considered that my morning ablutions were performed too early. Often after a sleepless night I have been only too glad to have my bed made and myself refreshed by a nice wash, and even if a patient has to be awakened there are plenty of opportunities during the day in which the loss of sleep may be repaired. If patients were to be allowed to arrange a time-table for the performance of various duties I am afraid the order and comfort which are the prevailing features of the wards would be conspicuous by their absence.

When one considers the many benefits derived from the moment patients are received into the ward until the day they return to their homes restored to health and strength, it seems to me just a little ungrateful to find fault with any of the arrangements that may not meet with one's personal approbation. Although the correspondent of eleven years is apparently a bright little boy, his childish little grumble is hardly worth notice, and his remarks concerning the nurses who "look upon the washing of patients as a game" is too ridiculous for words; anyone who has been under the care of hospital nurses can testify (as I am pleased to be able to do) to the loving care with which they perform the most minute and menial duty that may conduce to the comfort of their patients.—Yours faithfully, A Grateful Patient.

